# Implicit motor imagery: examining motor vs. visual strategies in laterality judgments among older adults

**DOI:** 10.3389/fpsyg.2024.1445152

**Published:** 2024-10-02

**Authors:** Aneet Saran, Jonathan J. Marotta

**Affiliations:** Faculty of Arts, Department of Psychology, University of Manitoba, Winnipeg, MB, Canada

**Keywords:** motor simulation, hand laterality judgment task, implicit motor imagery, age, sex

## Abstract

Cognitive states like motor imagery (MI; simulating actions without overtly executing them) share a close correspondence with action execution, and hence, activate the motor system in a similar way. However, as people age, reduction in specific cognitive abilities like motor action simulation and action planning/prediction are commonly experienced. The present study examined the effect of visual–spatial processing for both typical and challenging upper-limb movements using the Hand Laterality Judgment Task (HLJT), in which participants were asked to judge whether the depicted hand is a left or right hand. Several main findings emerged: (1) Compared to younger adults, older adults exhibited slower responses and greater error rates in both Experiment 1 and 2. This suggests that visual–spatial transformations undergo alterations with age; (2) Older adults displayed higher error rates with realistic hands at both back and palm viewpoints of the hands compared to younger adults. However, this pattern did not hold for response times; (3) Participants responded faster to medial hand orientations (i.e., closer to the midline of the body) compared to lateral hand orientations (i.e., farther from the midline of the body) for palm-views in both Experiment 1 and Experiment 2. Given that we observed better performance on medial orientations compared to lateral orientations, this suggests that participants follow the same motor rules and biomechanical constraints of the represented movement. Novel information is provided about differences in individuals’ use of strategies (visual vs. motor imagery) to solve the HLJT for both mannequin and real hands.

## Introduction

Motor imagery is a cognitive process that allows for the rehearsal of movements without any motor output (Decety, 1996; Jeannerod and Decety, 1995; Jeannerod, 1994, 1997, 2001). Consider an athlete mentally preparing to shoot free-throws. The player must first simulate motor representations that correspond to the unfolding action being imagined. This enables the player to internally reproduce the action by shifting their body to an optimal position, aligning the shooting hand, and finally shooting the basketball. Simulating the free-throw movements involves a series of complex and interacting processes that obey the same motor rules and biomechanical constraints of the represented movement (Decety et al., 1989; Gentili et al., 2004; Jeannerod and Decety, 1995). According to the *Motor Simulation Theory* (Jeannerod, 2001), there appears to be an important relationship between executed and simulated actions. Cognitive states such as kinesthetic motor imagery share the same representations as their overt counterparts (Decety et al., 1989; Jeannerod and Decety, 1995; Jeannerod, 1994, 1997). In particular, this theory postulates that motor imagery activates the motor system in a way that is similar to what is observed during action execution. Despite the similar mechanisms involved in motor simulation and execution, imagery involves the complete inhibition of overt output (i.e., the descending pathways and spinal circuits that normally carry voluntary commands appear to be blocked, preventing motoneuron activation). In fact, previous research has reported similar temporal (Decety and Michel, 1989; Jeannerod, 1994; Papaxanthis et al., 2002; Saimpont et al., 2012), physiological (Decety et al., 1991; Decety et al., 1993; Paccalin and Jeannerod, 2000; Yue and Cole, 1992), and neural correlates between motor imagery and actual movement (Gerardin et al., 2000; Hanakawa et al., 2003; Jeannerod, 2001; Roth et al., 1996; Porro et al., 1996; Porro et al., 2000; Heuninckx et al., 2008).

### Implicit motor imagery during mental rotation of the hands

**Figure 1:**
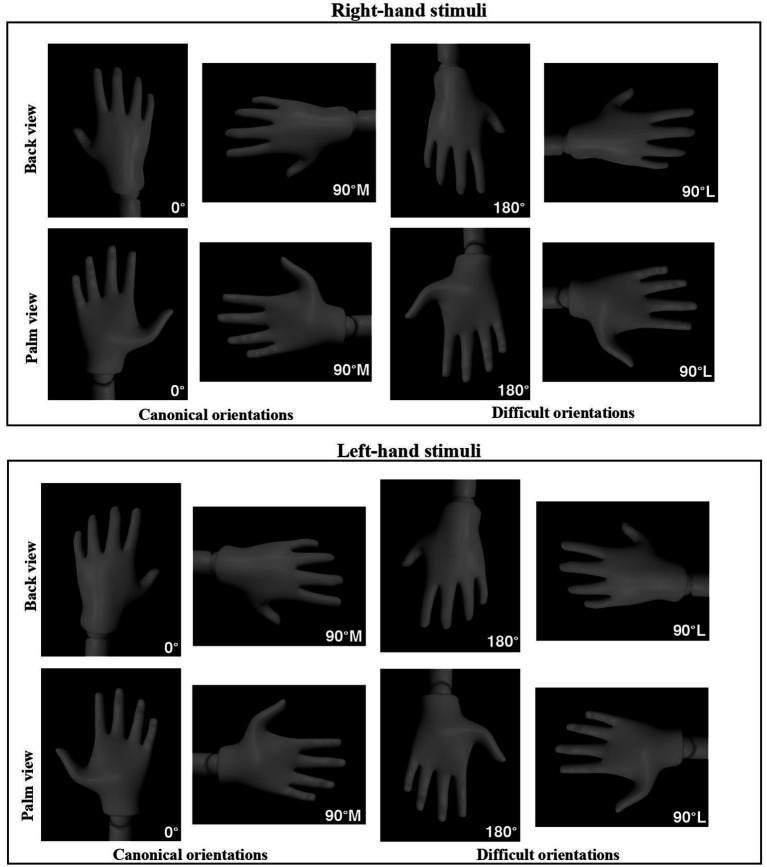
Right and left mannequin hand stimuli displayed from two different viewpoints (back and palm) and in four different orientations: 0°, 90° medial (canonical orientations), 90° lateral, and 180° (difficult orientations), used in the *Hand Laterality Judgment Task*.

The ability to spatially transform a mental image can be implicitly triggered when individuals unconsciously simulate an action. Implicit cognitive processes are also employed in the *Hand Laterality Judgment Task*, in which participants are asked to determine whether a depicted hand is right or left presented at different angles (Cooper and Shepard, 1975; Parsons, 1987; Parsons, 1994; Sekiyama, 1982; Figure 1). It’s thought that participants solve this task by mentally rotating their own hand into the orientation of the visually presented hand (Dalecki et al., 2012; Jeannerod and Decety, 1995; Parsons, 1994). Parsons (1994) and Parsons et al. (1995) conducted a series of experiments in which he asked participants to make hand judgments and found that the time taken to mentally rotate one’s hand is similar to the time taken to execute the corresponding movement. Unlike external objects (i.e., 3-D objects), the mental rotations of one’s own hand are strongly influenced by the same motor rules and anatomical constraints that shape real movements (Petit et al., 2003; Sekiyama, 1982). Consider the example of simulating a push up, in which one places their palms in an awkward orientation. It would be quite impractical and effortful to execute this motor act. Relative to motor execution, mental spatial transformation of hands presented at lateral (i.e., facing away from mid-sagittal plane of the body) and 180° (i.e., difficult orientations) require an increased angle of rotation resulting in longer recognition times and greater errors. While mental spatial transformations of hands at 0° and 90°M (i.e., simple orientations) lead to faster response times and fewer errors. Palm viewpoints typically result in prolonged response times and higher error rates at 90°L compared to 180° orientations (ter Horst et al., 2010; Bläsing et al., 2013). This suggests that individuals engage in longer rotational pathways when mentally rotating hands from 90°L to 0°, leading to non-linear patterns in response times. Conversely, medial orientations of hands (facing toward the mid-sagittal plane of the body) elicit the fastest and most accurate responses (Parsons, 1994), indicating shorter rotational pathways consistent with findings observed with 3-D objects (Cooper and Shepard, 1975). This notion of a medial-over-lateral advantage (MOLA) proposes close correspondence between certain kinematic and temporal characteristics of actual movements and their mental simulations (Bläsing et al., 2013; Dalecki et al., 2012; Parsons, 1994). There is even some evidence to suggest that individuals with chronic health conditions such as functional movement disorders (Navaratnam et al., 2021), Parkinson’s disease (Helmich et al., 2007; Scarpina et al., 2019; Bek et al., 2022), chronic arm/shoulder pain (Coslett et al., 2010), and focal hand dystonia (Fiorio et al., 2006) plan and prepare movements of their affected upper-limbs similarly to those of healthy controls.

### Kinesthetic motor vs. visual motor strategy

A laterality judgment task can be solved by using two imagery strategies, depending on the view of the hand: kinesthetic motor strategies (first-person perspective, imagining one’s own hands as if seen directly from one’s own viewpoint) and visual motor strategies (third-person perspective, visualizing someone else’s hands as if observing them externally; Bläsing et al., 2013; Gentilucci et al., 1998; Nagashima et al., 2021). In particular, visual motor imagery is the visual representation of an action (e.g., running on a treadmill) whereas kinesthetic motor imagery is the sensory experience of the motor act (e.g., feeling the glutes, hamstrings, and quads while running on a treadmill; Mizuguchi et al., 2015). Generally, one utilizes kinesthetic motor strategies when observing palm-hand stimuli, and visual motor strategies when observing back-hand stimuli (Bläsing et al., 2013; Gentilucci et al., 1998; Nagashima et al., 2021).

Palm views appear to exhibit more anatomical constraints than back views (Bläsing et al., 2013; Cooper and Shepard, 1975; Ionta et al., 2007; Parsons, 1987; Parsons et al., 1995; Sekiyama, 1982). Functional Magnetic Resonance Imaging (fMRI) data suggests that palm-view stimuli are processed by similar brain regions involved in motor simulation and execution, while back-view stimuli are thought to activate visual areas of the brain (Zapparoli et al., 2014). This supposed shift in strategy offers that when viewing back hand stimuli, participants employ visual motor strategies, whereas palm-hand stimuli employ motoric strategies (Bläsing et al., 2013; Gentilucci et al., 1998; Nagashima et al., 2021). The use of motoric strategies when solving laterality judgment tasks is further supported by patient studies (Rumiati et al., 2001; Sekiyama, 1982; Tomasino et al., 2003). For example, patients with left hemisphere damage (i.e., left parietal cortex) show impaired visual–spatial transformation of hands presented from the first-person perspective, but mental simulation of 3-D objects remains intact (Rumiati et al., 2001). Those with damage to the right hemisphere, however, have intact mental simulations of the hand despite impaired external object simulations (Tomasino et al., 2003). This strong dissociation makes intuitive sense, as mentally rotating objects relative to the environment (object-centered reference) does not adhere to same anatomical constraints of real movements (Howard, 1982).

To determine the best strategy for solving the laterality judgment task, a participant’s visual and sensorimotor familiarity with the stimuli must be taken into account. Typically, we have more visual experience with the back of our hands than our palms. For example, when grasping an object, we tend to focus more on the back of our hands than our palms, even though the typical method involves using the palm of our hands. Further evidence is provided by those with unilateral amelia (i.e., individuals born with only one hand; Funk and Brugger, 2008). To indicate laterality, unilaterally amelic patients pressed either the right or left key on a special keyboard using their preserved hand or stump. Participants showed faster laterality judgment times, particularly for the back view, when evaluating an image to their preserved hand than to their missing hand. This implies that visual and sensorimotor familiarity might play a role in influencing laterality judgments.

View is not the only variable influencing imagined spatial transformations of limbs; laterality is also significant (Cheng et al., 2020; Ionta et al., 2007; Ionta and Blanke, 2009; Parsons, 1987). Right-handed participants, who show a left-hemispheric dominance for motor control (Tomasino et al., 2003) recognize right hands faster, whereas left-handed participants do not show a left-hand preference (Gentilucci et al., 1998). Additionally, individual differences can influence laterality differences (Bobrova et al., 2021). For instance, the phenomenon of left–right confusion, where some individuals have difficulty distinguishing between left and right sides of their bodies, may contribute to the differences observed in the HLJT (Ocklenburg et al., 2011; van der Ham et al., 2021).

### Age-related differences in motor imagery

As people age, reductions in specific cognitive abilities like motor action simulation (Saimpont et al., 2009; Skoura et al., 2005) and action planning/prediction (Gabbard et al., 2011; Personnier et al., 2008; Skoura et al., 2008) are commonly experienced. There are now several examples of mental imagery tasks involving mental rotation of 3-D objects (Puglisi and Morrell, 1986), alphabetical letters (Cerella et al., 1981; Dror and Kosslyn, 1994; Iachini et al., 2019), and human faces (Adduri and Marotta, 2009; Habak et al., 2008) that have shown older adults to be slower and/or less accurate than younger adults. This outcome May relate to the idea that specific components of imagery such as image generation (i.e., ability to form a mental image) and image manipulation (i.e., the ability to spatially transform a mental image) are deteriorated in older adults (Briggs et al., 1999; Craik and Dirkx, 1992; Dror and Kosslyn, 1994; Schott, 2012). A similar age-related slowing and decline in accuracy has also been well-documented in motor imagery tasks (Beauchet et al., 2010; Gabbard and Cordova, 2013; Mulder et al., 2007; Personnier et al., 2010; Skoura et al., 2005; Saimpont et al., 2015; Zapparoli et al., 2014). When imagining parts of the body (i.e., hands), the ability to implicitly simulate complex upper-limb movements declines with aging (De Simone et al., 2013; Iachini et al., 2019; Saimpont et al., 2009; Saimpont et al., 2013; Sapsford et al., 2016). Consistent with the motor simulation hypothesis, biomechanical constraints normally applied during real movements affect visual–spatial transformation of hands in aging populations (Personnier et al., 2008; Saimpont et al., 2009).

### Sex differences in mental rotation of the hand

While sex differences in the mental rotation of objects have been widely acknowledged (Campos, 2014; Parsons et al., 2004; Linn and Petersen, 1985), studies of implicit motor imagery have not yielded conclusive results. Using the classical version of HLJT, previous literature has shown that men judge left-handed stimuli faster than women (Mochizuki et al., 2019). Moreover, sex differences have been found when judging hand laterality of right and left images from different views (Conson et al., 2020). For instance, males exhibit faster response times when viewing palm hand stimuli at 0°, 90°, and 180° orientations, while women display faster response times when viewing right back hands at 0° orientation and left back hands at 0° and 90° orientations (Conson et al., 2020). Consequently, males and females use different motor simulation strategies depending on which side of the hand is being shown, with males mainly using kinesthetic motor strategies for palm-view stimuli and females using visual strategies for processing back-hand stimuli. The above-cited literature on this topic reveals sex differences in implicit motor imagery use between males and females.

The present study compared implicit motor simulations between older and younger adults using the HLJT. Given the popularity of this task in measuring implicit motor simulations, two experiments were conducted using different hand stimuli. The first experiment utilized mannequin hands, while the second experiment employed realistic hands to determine if both stimuli elicit similar patterns of behavioral responses in younger and older adults. In view of well-documented declines in sensorimotor and cognitive functions in aging populations, it was hypothesized that older adults will be less accurate and have slower reaction times than young adults in their laterality judgments when presented with mannequin and realistic hand stimuli in both simple and difficult orientations. As males and females exploit motor simulation processes differently, it was anticipated that older women will perform better on stimuli presented from the back, while older men will perform better on palm-view stimuli. Overall, simulated hand movements were expected to obey the same motor rules and biomechanical constraints that govern real-world hand movements.

## Experiment 1: assessing hand laterality with mannequin hands

### Methods

#### Participants

Forty young adults (20 female, 20 male; age range 17–37 years old; *M* = 22, *SD* = 5.05) were recruited from the University of Manitoba’s psychology participant research pool and received course credit for their participation. Forty older adults (24 female, 16 male; age range 65–94 years old; *M* = 76.54, *SD* = 7.25) were recruited through local newsletters, word of mouth, talks presented at independent living facilities, and finally from the Centre on Aging’s database at the University of Manitoba. Conducting a power analysis using G*Power version 3.1 (Faul et al., 2009), the aim was to achieve a power of 0.80 in order to detect a medium effect size of 0.3, at a significance level of *α* = 0.05. The analysis suggested a total of 68 participants for a mixed Analysis of Variance (ANOVA); however, data from 80 participants were collected. Prior to participation, all participants provided online informed consent. All participants had normal or corrected to normal vision and were right-hand dominant as determined by a modified version of the Edinburgh Handedness Inventory (Oldfield, 1971). The EDI scores ranged from 7 to 9, with a mean score of 8.89 (SD = 0.35). All participants engaged in regular physical exercise, cognitive activities, (e.g., reading books, doing puzzles, etc.) and had no known neurological problems. On average, younger adults participated in physical activities for 4 days a week and engaged in cognitive activities for 5 days. Alternatively, older individuals exercised 5 days a week and engaged in cognitive tasks for 7 days a week. Participants over the age of 65 completed a modified version of the Mini Mental State Examination (MMSE; Folstein et al., 1975) and scored within normal limits (≥24). The MMSE scores ranged from 24–30, with a mean score of 28.55 (SD = 1.8). The simple reaction time (SRT), in which participants responded to stimuli with both hands was also measured (young: right = 288 ms [*SD* = 62.2], left = 291 ms [*SD* = 63.4]; old: right = 409 ms [*SD* = 70.2], left = 403 ms [*SD* = 67.5]). All procedures were approved by University of Manitoba Research Ethics Board, Fort Garry, our Faculty, the COVID Recovery Response Team, the COVID Recovery Steering Committee, and the University Provost. All procedures performed in studies involving human participants were in accordance with the ethical standards of the institutional and/or national research committee and with the 1964 Helsinki declaration and its later amendments or comparable ethical standards

#### Stimuli and materials

The task was created using lab.js, an open-source online experimental platform for behavioral and cognitive sciences (Henninger et al., 2022). Participants were shown gray-colored depictions of left or right mannequin hands measuring 578 × 447 pixels, created with Poser 4.0 software (Curious Labs, Santa Cruz, CA, USA). To ensure a universal and inclusive design, gray-colored mannequin hands were used. The hand-stimuli were created in accordance with previous studies that used realistic animated hands (Saimpont et al., 2009) and black-and-white hand drawings (Dalecki et al., 2012; Iachini et al., 2019; Parsons, 1987, 1994). This study, however, excluded certain hand features (e.g., hand creases and nails) and instead used a combination of lighting and shading to highlight significant features of the hand in order to differentiate between two viewpoints.

Target hand images were presented one at a time on a black background, measuring 800 × 600 pixels. Participants were shown right or left hands, displayed from one of two different viewpoints (back or palm) and in one of four different orientations: 0° = upward position, 90° medial = facing toward the midsagittal plane of the body, 90° lateral = facing away from the midsagittal plane of the body, and 180° = downward position (Figure 1). Orientations were selected based on previous research (Bläsing et al., 2013; Conson et al., 2012; Conson et al., 2020, Funk and Brugger, 2008; Ionta et al., 2012; Saimpont et al., 2009) where rotation angles were increased by 90°. To indicate the laterality of left- and right-hand images, participants pressed the ‘K’ key for right-hand stimuli with their right hand and the ‘A’ key for left-hand stimuli with their left hand. The experiment was divided into 3 series, each consisting of 32 stimuli presented in random order. A total of 96 trials (2 [left- and right-hand images] × 2 [palm and back views] × 4 [0, 90° medial, 90° lateral, and 180°orientation] × 6 trials per unique stimuli type) were administered to each participant. The task programmed in lab.js was hosted on the online platform Github.

#### Procedure

##### Young adults

In accordance with COVID-19 restrictions, data collection involving younger adults was conducted remotely. Prior to beginning the experiment, participants were instructed to complete a variety of demographic questions followed by the SRT task. In the SRT task, participants were instructed to focus on a fixation cross (displayed for 1,500 ms) and press the space key with their right or left hand when they saw a red circle. A total of ten trials were performed for each hand. Participants completed two training phases to familiarize themselves with the experimental protocol. Similar training phases were used in a previous normal aging study reported by Saimpont et al. (2009). In training phase one, 16 unique hand stimuli were presented on the screen to ensure all participants could physically move and match their hands to the stimuli. The instructions provided real hand images that matched the experimental hand stimuli to clarify the task. Participants pressed the ‘Y’ key to indicate their ability to physically move and match their hand to the image displayed on the screen, and the ‘N’ key if they were unable to do so. Participants unable to physically move and match their hand to the screen image were excluded from the analysis. Each trial began with a white fixation cross appearing on the computer screen for 1,500 ms, followed by a target hand image that remained on the screen until the participants physically attempted to move and match their hand to the orientation and view of the hand-stimuli.

The second training phase was designed to familiarize participants with left-and-right hand images presented in different orientations and views. In this training phase, participants were asked to rest their hands palm-down on the keyboard. Participants completed 32 practice trials in this training phase to ensure they were performing the trials according to the instructions. Participants did not physically move and match their hands to the image displayed on the screen. Instead, participants selected the ‘K’ key for right hand-stimuli and the ‘A’ key for left hand-stimuli. The trials were interspersed with a white fixation cross (displayed for 1,500 ms), and a target image remained on the screen until the participants indicated the laterality by either pressing either the left or right button on the keyboard.

Once the trial ended, participants were shown a screen to prepare them for the next trial. Once both training phases were complete, participants began the experimental phase. The experimental phase included the same instructions provided in phase two, except participants were instructed to respond as quickly and accurately as possible. Upon finishing the experiment, participants were directed to the debriefing form.

##### Older adults

As COVID-19 restrictions eased slightly, permission was sought to test older adults in-person to ensure minimal loss of data. Detailed information regarding the study was provided through phone calls and emails to participants. Data collection appointments for in-person data collection were sent to interested participants. On the day of the visit, a laptop with the experiment was set up in the participant’s home. A link to the experiment was emailed to participants who chose to use their personal desktop or laptop computer. The researcher monitored participants’ motor movements during training phase one, which required them to move and match their hands to stimuli. For training phase two, researchers made sure that participants utilized kinesthetic motor strategies by pressing either the A key (left-hand stimuli) or the K key (right-hand stimuli) instead of physically moving their hands. After the training phases, the experimental phase began, which was similar to the virtual condition. During this phase, the researcher reminded participants not to physically move and match their hands to the hand-stimuli presented and respond as quickly and accurately as possible.

#### Data analyses

The goal of the present study was to examine the effects of implicit motor imagery on aging using the *Hand Laterality Judgment Task*. Analyses were mainly concerned with response times (RTs) and accuracy. RTs were defined as the interval between the onset of a hand stimulus on the screen to the push of either an ‘A’ or ‘K’ response button; Accuracy was defined as the number of correct responses out of the total number of trials. With accuracy treated as count data, the proportion of errors was calculated for each unique condition based on 6 trials. Scores of 6 indicated 0% proportion of errors (i.e., 100% accuracy).

Trial data within each condition was averaged to create mean condition values for each participant. When trials were missing for participants, the data were substituted with the mean for that condition, if applicable. Response times for correct trials were analyzed with a 2 (Sex: Male vs. Female; between-subjects) × 2 (Age: Young vs., Old; between-subjects) × 2 (Laterality: Left vs. Right; within-subjects) × 2 (View: Back vs. Palm; within-subjects) × 4 (Orientation: 0° vs. 90° Lateral vs. 90° Medial vs. 180°; within-subjects) mixed Analysis of Variance (ANOVA) using *jamovi* (Version 1.6). Any violations of sphericity were tested for using Mauchly’s test and were addressed using a Greenhouse–Geisser correction. When interactions were present, post-hoc pair-wise comparisons were carried out using a Bonferroni correction. Accuracy was analyzed using a Generalized Linear Mixed Model (GLMM) in jamovi (Version 1.6), assuming a Poisson distribution for count data with a log link function. Different models were fitted and tested using different combinations of fixed effects, followed by the removal of non-significant predictor variables (in this case, the laterality predictor variable was non-significant and therefore removed). The final model included sex, age, orientation, and view as fixed effects, while participant ID was treated as a random effect to control for the influence of between-participants variation. The following GLMM was used to fit the data: Accuracy~1+ Sex + Age + Orientation+ View + (1|Participant ID). When interactions were present, post-hoc pair-wise comparisons were carried out using a Bonferroni correction.

### Results

All participants were required to mentally simulate upper-limb movements without any overt movements in order to determine response time and accuracy. Each participant’s mean RTs were calculated for each condition and entered into a repeated measures ANOVA. Additionally, mean proportions of errors were calculated for each condition and entered into a GLMM.

Age-related differences in response time and accuracy data were hypothesized, with older adults being less accurate and having slower reaction times than younger adults when judging laterality from canonical and difficult hand orientations. Moreover, older women were hypothesized to perform better on hand stimuli presented from the back, whereas older men were hypothesized to perform better on hand stimuli presented from the palm-view. Lastly, it was hypothesized that simulated hand movements would follow the same motor rules and biomechanical constraints of real-world hand movements.

## Excluded data

A total of 9 older adults were excluded from analysis of both response time and accuracy due to their error rates surpassing 30%, as determined by the overall raw data for their accuracy scores. Trials with durations below 500 ms or above 7,500 ms were removed, and the same trials were excluded from the accuracy analysis. Additionally, any trials involving incorrect responses during the laterality judgment task were also excluded. A total of 12.75% of the trials were excluded from the analysis. A total of 31 older adults were included in the analysis (18 female, 13 male; age range 65–94 years old; *M* = 76.88, *SD* = 7.39).

### Response times

#### Main effects

A significant main effect of View was found when determining the laterality of right- and left-hand images, *F*(1, 67) = 52.21, *p* < 0.001, η_p_^2^ = 0.438. Hands viewed from the back had faster response times than those viewed from the palm. A significant main effect of Laterality was found, *F*(1, 67) = 9.28, *p* = 0.003, η_p_^2^ = 0.122. Participants had faster response times to right than left hands, reflecting a preference for their dominant hand. A significant main effect of Orientation was found, *F*(2.50, 157.83) = 167.21, *p* < 0.001, η_p_^2^ = 0.649. Response times were faster for canonical orientations and slower for difficult orientations. Finally, a significant main effect of Age was found, *F*(1, 67) = 90.28, *p* < 0.001, η_p_^2^ = 0.574. Younger adults had faster response times than their older counterparts, as hypothesized.

**Figure 2:**
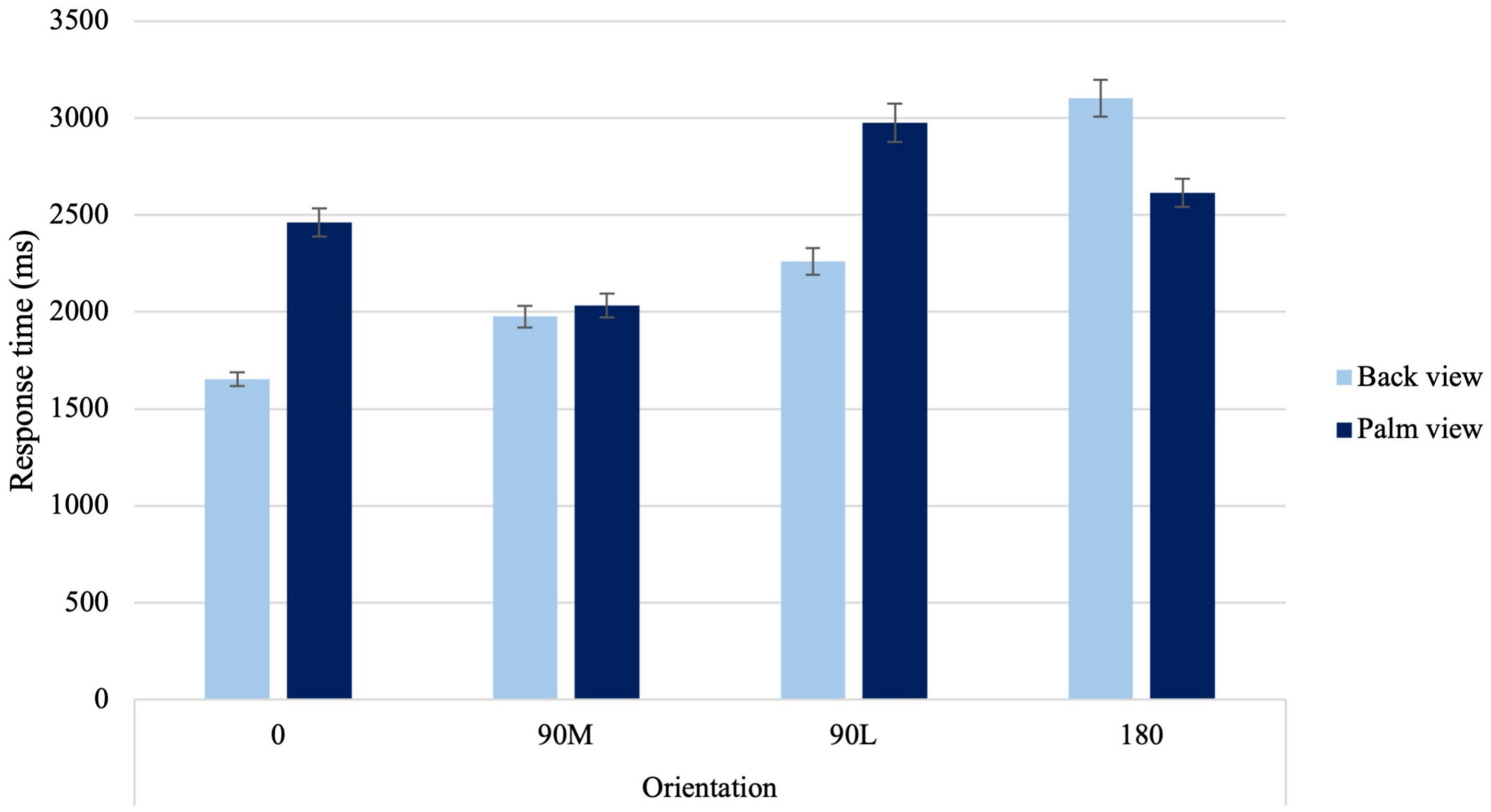
Average response time to the four different orientations for back and palm views. *Error bars* represent standard error of the means.

A significant Laterality × Sex Condition interaction was found, *F*(1, 67) = 6.73, *p* = 0.012, η_p_^2^ = 0.09. When judging the laterality of hands, males had faster response times to right than left hands (*p* = 0.002). A significant Laterality × Age Condition interaction was found, *F*(1, 67) = 4.97, *p* = 0.029, η_p_^2^ = 0.069. Younger adults had faster response times to right- and left-hands than their older counterparts (*p* < 0.001). In addition, older adults exhibited faster response times to their dominant right hand compared to their non-dominant left hand (*p* = 0.006). Lastly, a significant View × Orientation Condition interaction was found, *F*(2.27,151.86) = 71.21, *p* < 0.001, η_p_^2^ = 0.515 (Figure 2). When viewing the back of the hand, canonical orientations (0° and 90°M, p < 0.001) resulted in faster response times and difficult orientations (90°L and 180°, *p* < 0.001) resulted in slower response times. For hand simulations at 90°M palm, response times were the fastest compared to difficult orientations (90°L and 180), suggesting a MOLA. Additionally, hand simulations at 0° were equivalent to those at 180° palm.

### Accuracy

#### Main effects

In examining the frequencies of errors participants made when indicating the laterality of right-and left-hand images, a significant main effect of View was found, χ^2^ (1) = 15.1, *p* < 0.001. Higher proportion of errors occurred for palm than back views. A significant main effect of Orientation was found, χ^2^ (3) = 47.87, *p* < 0.001. Higher proportion of errors occurred for difficult (90°L and 180°) than canonical orientations (0° and 90°M). Furthermore, a significant main effect of Age was found, χ^2^(2) = 117.37, *p* < 0.001. Higher proportion of errors occurred for older than younger adults.

**Figure 3:**
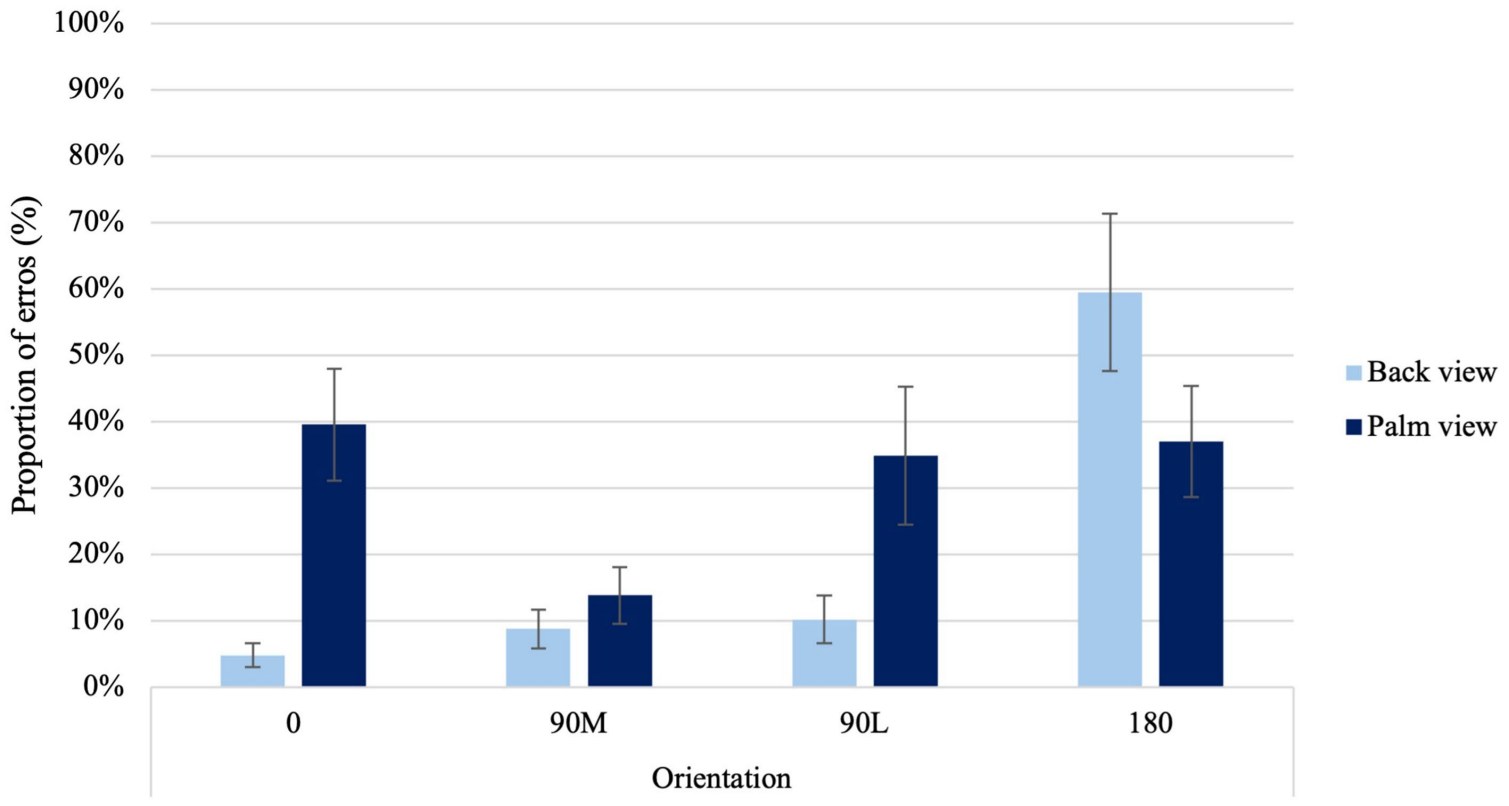
Average proportion of errors at the four different orientations for back and palm views. *Error bars* represent standard error of the means.

A significant View × Orientation Condition interaction was found, χ^2^ (3) = 69.66, *p* < 0.001 (Figure 3). In contrast to the back view of the hand, palm views showed higher errors at 0° than at 90°M, suggesting a MOLA effect (*p* < 0.001). Additionally, when palm views of 0° and 90°L orientations were compared with back views of the same orientations, higher error proportions were observed (*p* < 0.001). In line with the hypothesis, this result indicates that accuracy rates observed reflect similar motor rules and biomechanical constraints that govern real movements.

### Discussion

#### Orientation and view effects on laterality judgments

Participants placed a greater emphasis on motor imagery when viewing palm-view stimuli, as evidenced by an overall increase in response times and errors. This suggests that participants may engage in additional cognitive resources, such as simulating the physical constraints of real movements, which may increase the cognitive load, resulting in slower response times and more errors as compared to a visual strategy that may require less cognitive resources. Additionally, palm-views demonstrated a MOLA with a non-linear pattern in the behavioral measures. Prolonged response times and higher errors rates were observed when presented with 90°L, as opposed to 180° orientations (ter Horst et al., 2010; Bläsing et al., 2013). This suggests that they were following a longer rotational pathway. In contrast, when attempting to judge the laterality of right-and-left hands from the back view, participants primarily used visual strategies, resulting in a noticeable linear increase in behavioral measures. This suggests that participants were following shorter rotational pathways, in line with findings observed with 3-D objects (Cooper and Shepard, 1975). For instance, longer response times and higher errors rates were observed when presented with 180°, as opposed to 90°L orientations (ter Horst et al., 2010; Bläsing et al., 2013).

#### Age-related declines in implicit motor imagery

In terms of solving the HLJT, participants utilized both motor and visual strategies depending on the view and orientation of the experimental hand stimuli. As hypothesized, older adults performed significantly worse. The results are in support of previous research (De Simone et al., 2013; Hernández-Guillén et al., 2021; Saimpont et al., 2009), in that older adults show a more pronounced decline in their ability to simulate certain upper-limb movements. When compared to younger adults, older adults generally had slower response times to right- and left-hands, particularly for their non-dominant hand. A similar age-related decline for the non-dominant hand has been observed in previous studies (Saimpont et al., 2009). The results from this study also showed a slower response to typical and challenging hand orientations for right- and left-hands. Cognitive functions, such as speed of processing (i.e., the speed at which a cognitive task is completed), tend to decline with age (Salthouse, 1979, 1980, 1996). There has been research showing that older adults have slower response times for determining body-part laterality, including the hands (Hernández-Guillén et al., 2021). Considering this, older adults may take longer to make a decision about the laterality of hands than young adults. An alternative explanation for this age-related decline in RT may be due to the nature of the HLJT. Previous research has shown that there is an association between aging and delayed choice reaction time (CRT) tasks (Woods et al., 2015; Dykiert et al., 2012), particularly when preparatory intervals are present (i.e., the time between the beginning of a trial and the presentation of the stimulus; Hardwick et al., 2022). Considering this was a two-choice response task with a preparatory interval between responses (i.e., either a ‘A’ key response for left-hand stimuli or the ‘K’ key response for right-hand stimuli with a “get ready” interval between trials), older adults may have taken longer to respond to the varying hand-stimuli in this task.

#### Sex differences in motor simulation processes

Males responded faster to right hands than left hands, whereas the same was not observed for females. When solving the HLJT, males may show a preference for their visual and sensorimotor familiar dominant hand. In a similar study, it was reported that males respond faster when viewing right hands from the back (Conson et al., 2020). However, other studies have reported no differences between men’s responses to left- and right-hands (Mochizuki et al., 2019). Further research is still needed to examine sex differences when determining the role of hand laterality in this task.

## Experiment 2: assessing hand laterality with real hands

The results of Experiment 1 confirmed the hypothesis that older adults exhibited slower reaction times in comparison to younger adults when judging hand laterality. Furthermore, age-related differences in accuracy were observed, with older adults making a greater number of errors as opposed to their younger counterparts. However, the high error rates for the back view (i.e., close to 60%) and palm view (ranging from 35 to 40%) could indicate a challenge in distinguishing between the back and palm views of the mannequin hands. Given the high error rates, Experiment 2 employed real hands for the *Hand Laterality Judgment Task* (HLJT). The decision was made because it is common to rely on certain hand features to determine the back or the palm of our hands, such as the unique hand creases or the nails. It was hypothesized that error rates for both back and palm view conditions, presented in both canonical and difficult orientations would decrease. Furthermore, it was hypothesized that age-related patterns in response time and accuracy would remain consistent with those observed in Experiment 1; specifically older adults were expected to be less accurate and have slower response times compared to their younger counterparts when making laterality judgments from different viewpoints and orientations.

### Methods

#### Participants

Twenty young adults (11 female, 9 male; age range 17–29 years old; *M* = 21.68, *SD* = 3.9) were recruited from the University of Manitoba’s psychology participant research pool and received course credit for their participation. Twenty one older adults (16 female, 5 male; age range 65–93 years old; *M* = 76.81, *SD* = 8.16) were recruited through local newsletters, word of mouth, talks presented at independent living facilities, and finally from the Centre on Aging’s database at the University of Manitoba. An *a priori* power analysis using G*Power version 3.1 (Faul et al., 2009) was used, aiming for a power of 0.80 to detect a medium effect size of 0.35, at a significance level of *α* = 0.05. The analysis suggested a total of 38 participants for a mixed Analysis of Variance (ANOVA); however, data from 41 participants were collected. Prior to participation, all participants provided online informed consent. All participants had normal or corrected to normal vision and were right-hand dominant as determined by a modified version of the Edinburgh Handedness Inventory (Oldfield, 1971). The EDI scores ranged from 8 to 9, with a mean score of 8.95 (SD = 0.22). All participants engaged in regular physical exercise, cognitive activities, (e.g., reading books, doing puzzles, etc.) and had no known neurological problems. On average, younger adults participated in physical activities for 3 days a week and engaged in cognitive activities for 4 days. Alternatively, older individuals exercised 5 days a week and engaged in cognitive tasks for 6 days a week. Participants over the age of 65 completed a modified version of the Mini Mental State Examination (MMSE; Folstein et al., 1975) and scored within normal limits (≥ 24). The MMSE scores ranged from 24 to 30, with a mean score of 29.84 (SD = 0.50). The simple reaction time (SRT), in which participants responded to stimuli with both hands was also measured (young: right = 326.7 ms [*SD* = 151.85], left = 337.19 ms [*SD* = 164.37]; old: right = 506 ms [*SD* = 264.37], left = 489.65 ms [*SD* = 204.38]). All procedures were approved by University of Manitoba Research Ethics Board, Fort Garry, our Faculty, the COVID Recovery Response Team, the COVID Recovery Steering Committee, and the University Provost. All procedures performed in studies involving human participants were in accordance with the ethical standards of the institutional and/or national research committee and with the 1964 Helsinki declaration and its later amendments or comparable ethical standards

#### Stimuli, materials, and procedure

**Figure 4:**
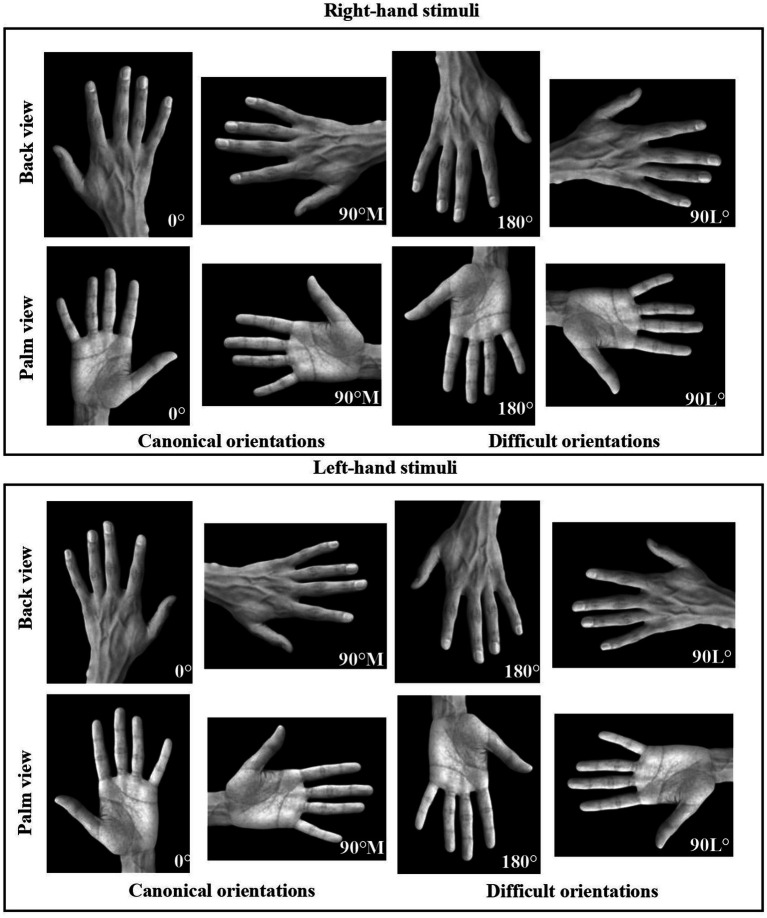
Right and left greyscale real hand stimuli displayed from two different viewpoints (back and palm) and in four different orientations: 0°, 90° medial (canonical orientations), 90° lateral, and 180° (difficult orientations), used in the *Hand Laterality Judgment Task*.

Participants were shown depictions of greyscale left or right real hands measuring 578 × 447 pixels (Figure 4). These images were taken with a high-quality camera in a home studio, using a black background drop. These hand stimuli ensured that all hand features, including creases and nails, were presented for participants to easily distinguish between the back and palm viewpoints. The remaining stimuli and materials were consistent with those outlined in Experiment 1. The procedure closely mirrored Experiment 1, with two notable differences. Firstly, all data for younger adults was collected in the *Neuropsychology of Vision: Perception and Action Lab*. Secondly, as COVID-19 restrictions were no longer in place during this phase of data collection, the precautions outlined in Experiment 1 were not utilized.

### Data analyses

Trial data within each condition was averaged to create mean condition values for each participant. When trials were missing for participants, the data were substituted with the mean for that condition, if applicable. Response times for correct trials were analyzed with a 2 (Age: Young vs., Old; between-subjects) × 2 (Laterality: Left vs. Right; within-subjects) × 2 (View: Back vs. Palm; within-subjects) × 4 (Orientation: 0° vs. 90° Lateral vs. 90° Medial vs. 180°; within-subjects) mixed Analysis of Covariance (ANCOVA) using *jamovi* (Version 1.6). Simple reaction time (SRT) was used as a covariate in the analysis. The Paired-Samples *T* Test procedure was used to examine the SRT differences between the right and left hands for both younger and older adults separately. Since no disparities in SRT were detected for either hand, averages of both right and left SRT were included as a covariate in the RT analysis. Sex was no longer employed as a between-subjects factor because obtaining an equal sample size for a special population (i.e., older adults) was not possible. As in Experiment 1, any violations of sphericity were tested for using Mauchly’s test and were addressed using a Greenhouse–Geisser correction. When interactions were present, post-hoc pair-wise comparisons were carried out using a Bonferroni correction. As in Experiment 1, accuracy was analyzed using a Generalized Linear Mixed Model (GLMM) in jamovi (Version 1.6), assuming a Poisson distribution for count data with a log link function. Different models were fitted and tested using different combinations of fixed effects, followed by the removal of non-significant predictor variables (in this case, the laterality predictor variable was non-significant and therefore removed), mirroring the approach taken in Experiment 1. The final model included age, orientation, and view as fixed effects, while participant ID was treated as a random effect to control for the influence of between-participants variation. The following GLMM was used to fit the data: Accuracy~1+ Age + Orientation+ View + (1|Participant ID). When interactions were present, post-hoc pair-wise comparisons were carried out using a Bonferroni correction.

### Results

With the use of real hand images, it was hypothesized that response time and accuracy patterns for older adults would closely resemble those seen in Experiment 1, apart from a significant decrease in error rates for both back and palm views. As in Experiment 1, it was hypothesized that simulated hand movements would adhere to the same motor rules and biomechanical constraints as real-world hand movements.

#### Excluded data

A total of 2 older adults were excluded from analysis of both response time and accuracy due to their error rates surpassing 30%, as determined by the overall raw data for their accuracy scores. Trials with durations below 500 ms or above 7,500 ms were removed, and the same trials were also excluded from the accuracy analysis. Additionally, any trials involving incorrect responses during the laterality judgment task were also excluded. A total of 10.1% of trials were excluded from the analysis. A total of 19 older adults were included in the analysis (14 female, 5 male; age range 65–93 years old; *M* = 76.84, *SD* = 8.52).

#### Response times

##### Main effects

A significant main effect of Orientation was found when determining the laterality of right- and left-hand images, *F*(2.32, 83.38) = 9.66, *p* < 0.001, η_p_^2^ = 0.212. In line with Experiment 1, we found quicker response times for canonical orientations, contrasted with slower response times for challenging orientations (*p* < 0.001). In addition, a significant main effect of the Simple Reaction Time (SRT) covariate was found, *F*(1, 36) = 6.5, *p* = 0.015, η_p_^2^ = 0.153. Older adults had slower reaction times in the SRT task compared to their younger counterparts.

##### Lower-order interactions

**Figure 5:**
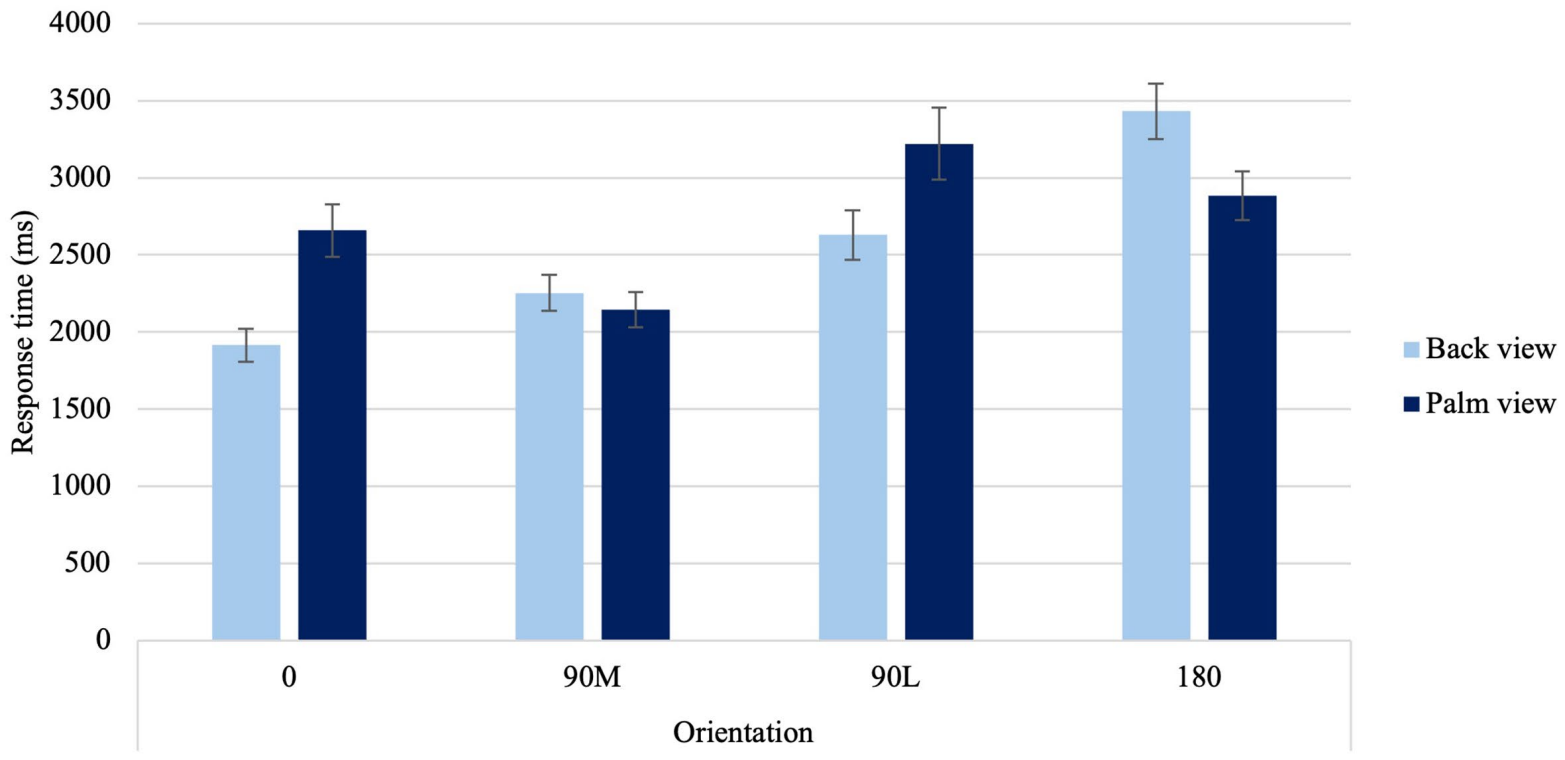
Average response times at the four different orientations for back and palm views. *Error bars* represent standard error of the means.

A significant View × Orientation Condition interaction was found, *F*(1.95, 70.12) = 6.94, *p* = 0.002, η_p_^2^ = 0.162 (Figure 5). When viewing the back of the hand, faster response times occurred for canonical orientations (0° and 90°M, *p* < 0.001) in comparison to difficult orientations (90°L and 180°, *p* < 0.001). Furthermore, significant differences were noted between canonical orientations and difficult orientations, with the fastest response times occurring at 0° and slowest response times occurring at 180 (*p* < 0.001). When viewing the palm of the hand, fastest response times occurred at 90°M, while the slowest response times occurred at difficult orientations (90°L), providing support a medial over lateral advantage (MOLA; *p* < 0.001).

##### Higher-order interactions

**Figure 6:**
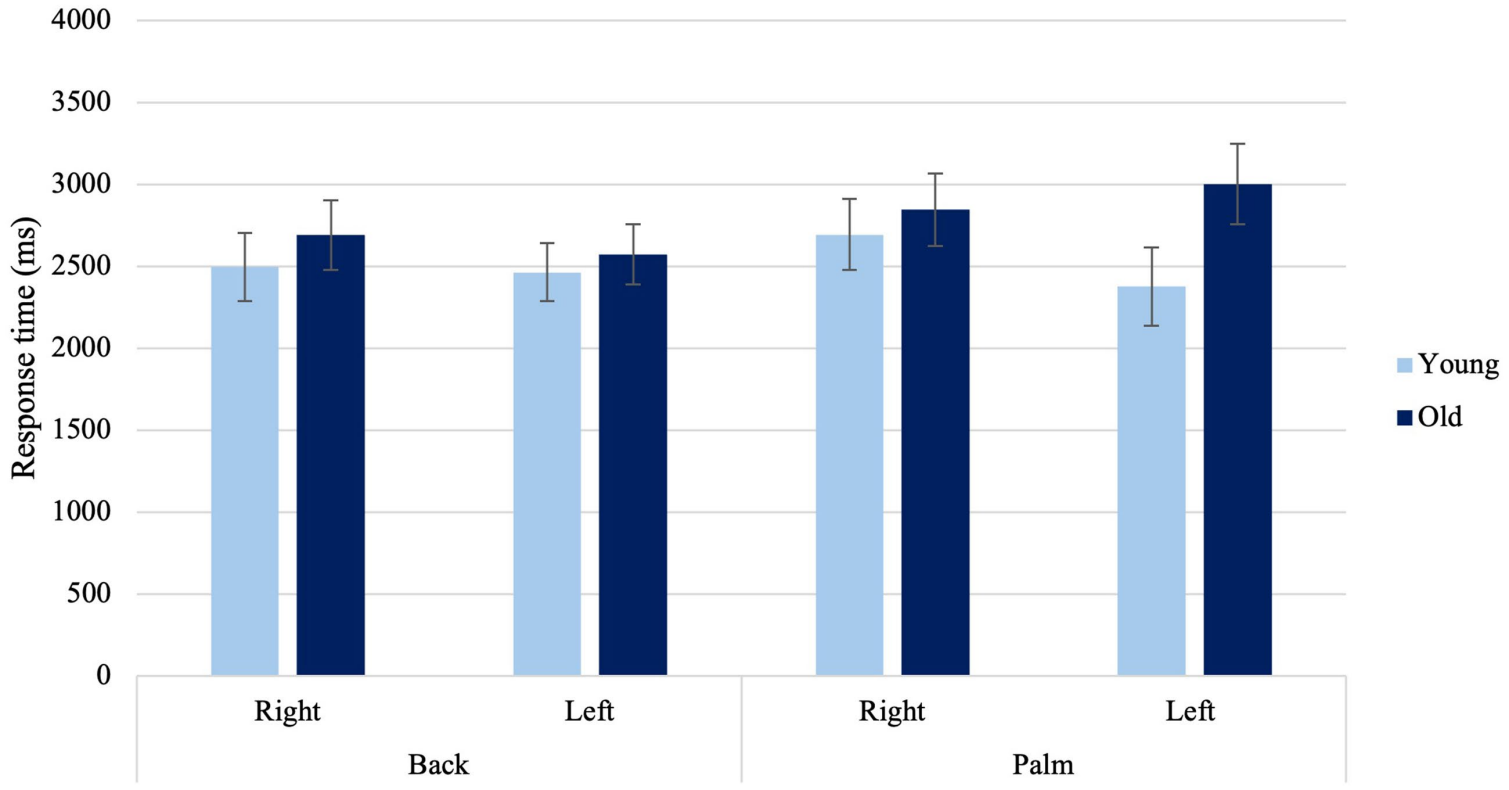
Average response times to back and palm views for right and left hands among younger and older adults. *Error bars* represent standard error of the means.

A significant Laterality × View × Age Condition was found when determining the laterality of right- and left-hand images, *F*(1, 36) = 4.36, *p* = 0.044, η_p_^2^ = 0.108 (Figure 6). Older adults exhibited faster response times when presented with left hands from the back view compared to left hands from the palm view (*p* = 0.025) and right hands from the palm (*p* = 0.016). Lastly, younger adults showed faster response times with their left hand when presented with hands viewed from the back, compared to when presented with the palm view of the right hand. (*p* = 0.035).

#### Accuracy

##### Main effects

A significant main effect of View was found, χ^2^ (1) = 5.84, *p* = 0.016, with higher proportion of error rates for palm over back views of the hand. Furthermore, a significant main effect of Orientation was observed, χ2 (3) = 25.43, *p* < 0.001, indicating a higher proportion of errors for difficult (90°L and 180°) compared to canonical orientations (0° and 90°M). Lastly, a significant main effect of Age was found, χ^2^ (1) = 7.79, *p* = 0.005, indicating a higher proportion of errors in older adults compared to their younger counterparts.

**Figure 7:**
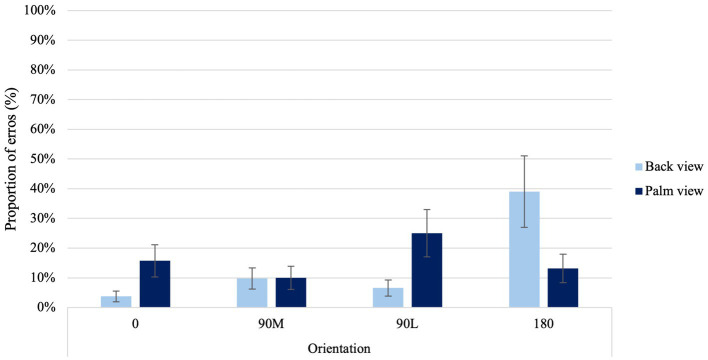
Average proportion of errors at the four different orientations for back and palm views. *Error bars* represent standard error of the means.

A significant View × Orientation Condition interaction was found, χ^2^(3) = 44.01, *p* < 0.001 (Figure 7). A higher proportion of errors occurred for the back 180° orientations compared to the canonical orientations (i.e., back 0° and 90 M°) and the 90 L° orientation from the same view (*p* < 0.001). When comparing palm views at 0° and 90°L with back views at the same orientations, higher error rates were observed, except for the 180° palm view, where higher error rates were noted for the back 180° orientation (*p* < 0.03). Moreover, elevated error rates were evident in the back 180° orientation in contrast to the palm 0° (*p* = 0.003) and 90 M° (*p* < 0.001) orientations. Additionally, a greater proportion of errors were noted in the palm 90 L° orientation compared to the back 0° (*p* < 0.001) and 90 M° (*p* = 0.023) orientations. A significant View × Age Condition interaction was found χ^2^ (1) = 3.91, *p* = 0.048. Older adults exhibited a greater proportion of errors when making laterality judgments from palms views compared to back views (*p* < 0.001). Additionally, older adults demonstrated a higher error rate in palm views compared to their younger counterparts, both in the palm view (*p* = 0.003) and back view (*p* = 0.002).

### Discussion

#### Orientation and view effects on laterality judgments

When viewing hands from the palm, a non-linear pattern of response times and error rates emerged, similar to what was observed in Experiment 1. This finding suggests that participants may have been employing a motor imagery strategy, by actively rotating their own hand to match the observed hand orientation (Shepard and Metzler, 1971; Parsons, 1994). In addition, the observed medial-over-lateral advantage (MOLA) for palm-view stimuli, where participants showed faster response times and fewer errors for medial orientations compared to lateral orientations, further supports the idea of a motoric strategy (ter Horst et al., 2010; Bläsing et al., 2013). Overall, this suggests that the mental simulations of hand movements align with physical mechanics of real-world actions. As in Experiment 1, the linear increase in response times and error rates for back-views suggests that participants were employing visual strategies to complete the *Hand Laterality Judgment Task* (HLJT). This supports the idea that mentally rotating hands becomes progressively more challenging as the angle of rotation increases (Cooper and Shepard, 1975).

#### Age-related declines in implicit motor imagery

When assessing the laterality of hand images, older adults exhibited a higher rate of errors at both viewpoints in contrast to younger adults, though this difference was not reflected in response times. The findings align with prior research indicating that older adults tend to demonstrate lower accuracy in the laterality judgment task (De Simone et al., 2013). During motor imagery, internal models are typically carried out with respect to the egocentric frame of reference, meaning individuals use their own perspective to simulate a hand movement (Wraga et al., 2005). These models effectively replicate the dynamic behavior of the human body (Jeannerod, 1997). When considering the use of an egocentric perspective with palm-view stimuli, it was observed that older adults demonstrated higher error rates. This finding mirrors previous research, which has shown a decrease in accuracy with age when employing an egocentric approach (Herman and Coyne, 1980; Devlin and Wilson, 2010). Previous research suggests that older adults’ accuracy in mental rotation tasks using an allocentric frame of reference (i.e., action simulation not centered around one’s perspective) is relatively intact (Inagaki et al., 2002). Drawing from this information, it seems that age-related differences may not have a considerable impact on visual imagery abilities, as evidenced by the greater accuracy observed for back-views.

Older adults responded faster when presented with left hands from the back view compared to left hands from the palm view and right hands from the palm. When making laterality judgments, both the viewpoint from which a hand is presented and the laterality of the hand itself can significantly influence the strategies individuals employ. Individuals generally have more visual experience with the back of their hands than with the palm, suggesting that as individuals age, they might increasingly depend on visual strategies, regardless of the hand’s laterality.

## General discussion

The present study explored age-related differences in implicit simulation processes using the *Hand Laterality Judgment Task* (HLJT). This study sought to determine (1) if biomechanical effects emerge for different views and orientations, (2) if similar strategies are used during implicit motor processes between younger and older adults, (3) if aging on its own impairs the ability to visually and spatially transform both typical and challenging hand movements. Considering the difference in response times and errors between typical and challenging hand movements, one can infer that the participants mentally rotated the experimental hand stimuli in a manner consistent with previous research (Parsons, 1994). When indicating the laterality of right-and-left hand images, response times and errors occurred in a manner consistent with the *Motor Simulation Theory* (Jeannerod, 2001). Furthermore, a predicted shift in strategy when viewing back hand stimuli (i.e., visual strategies) and palm-hand stimuli (i.e., motoric strategies) was observed.

### Orientation and view effects on laterality judgments

As hypothesized, performance on the HLJT declined as the complexity of orientation of the hand stimulus increased, regardless of the viewpoint (back vs. palm) or type of hand stimulus (mannequin vs. real hand). When observing the back of the hand, participants employed visual strategies for various reasons. First, response times and errors increased proportionally to the angle of rotation, as previously observed for 3-D objects (Cooper and Shepard, 1975). Second, a MOLA for the back view was not observed. This was evidenced by increased performance at 0° in contrast to 90 M°, and decreased performance at 180° as opposed to 90 L°. Third, as with external objects (Kolers and Pomerantz, 1971), participants followed the shortest rotational pathway when presented with back-view hand stimuli, as shown by their reduced response times and higher error rates for lateral than downright positions. In this case, hand properties for lateral movements were not accounted for, suggesting that visual strategies were used. In contrast, when viewing the hands from the palm, participants emphasized motor imagery for several reasons. First, response times and errors rates showed a non-linear pattern, particularly highlighted by a MOLA (ter Horst et al., 2010; Bläsing et al., 2013). In accordance with the previously reported MOLA (Cooper and Shepard, 1975; Parsons, 1994; Zapparoli et al., 2016), rotations made away from the body’s midline when compared with rotations made toward the body’s midline produced the greatest alterations to performance, verifying motor imagery mirrors hand mechanics of real movements. The fact that participants showed a greater increase in response times and errors when presented with palm views at 90°L, compared to 180° suggests that they were following a longer rotational pathway. As motor representations of the hand are more closely associated with first-person perspective, it is likely that participants May have used the configuration of their hand in space as a reference point to align their hand with the visually presented stimuli (Shepard and Metzler, 1971; Parsons, 1994). Consistent with previous research, when presented with orientations that required fewer rotations (i.e., 90°M), participants were able to simulate upper-limb movements more easily by aligning their own hands with the experimental stimuli. However, as the rotation angle increased (i.e., 90°L), the mental effort required to complete the task also increased (Shepard and Metzler, 1971; Parsons, 1994). A strategy such as this is indicative of participants obeying the same motor rules and biomechanical constraints of the represented movement during simulation. Overall, while direct comparisons between mannequin and real hands were not conducted, there was a notable view × orientation interaction reported for both, with similar patterns observed.

When employing specific strategies (motoric for palm views and visual for back-views), poorer performance was observed for mannequin hands, notably resulting in higher error rates. Considering the nature of the stimuli, it’s possible that participants resorted to a non-adaptive strategy in order to complete the HLJT. For example, errors rates of approximately 60% for back views presented at 180° were observed. If they exclusively relied on visual imagery—interpreting the palm view as the back of the hand—the observed high error rates would decrease. It appears that participants may have been more inclined to alternate between visual and kinesthetic motor strategies, indicating a potentially greater challenge for mannequin hand stimuli.

### Age-related differences in motor imagery

In general, a decrease in performance was observed in Experiment 1 and 2 for older adults compared to their younger counterparts. In comparison to younger counterparts, older adults displayed longer response times and a higher rate of errors when observing both mannequin and real hands, a pattern that echoes earlier research findings (De Simone et al., 2013; Iachini et al., 2019). As hypothesized, in Experiment 1, older adults exhibited slower responses with both their dominant and non-dominant hands compared to younger participants (Saimpont et al., 2009). Experiment 2 was conducted to further investigate the type of strategy older adults were employing to make laterality judgments. As older adults viewed realistic hands, their performance declined when presented with hand stimuli from both viewpoints. In particular, older adults showed higher error rates compared to their younger counterparts, whereas response times were comparable between the two groups.

Older adults demonstrated quicker responses to the back views of their non-dominant hand compared to palm views from both their right and left hands. Despite our hypothesis suggesting that older adults would respond faster to their dominant hand regardless of view, it appears that they found it easier to discern laterality from the back view with their non-dominant hand than from the palm view, for both their dominant and non-dominant hands. While these findings were unexpected, they may suggest that handedness plays a less significant role in laterality judgments for older adults than previously thought. Considering that palm-view stimuli employ motoric strategies, it is likely that older adults had greater difficulty judging the laterality of the hands from a palm view. The differences observed might also point to potential effects of left–right confusion, particularly in discerning the palm views, which could be an interesting area for further investigation. According to the results discussed above, as people age, reductions in specific cognitive abilities like motor action simulation (Saimpont et al., 2009; Skoura et al., 2005) are commonly experienced. Older adults May be compensating for their declining implicit motor imagery for laterality judgments by using visual compensation, as previously suggested by Zapparoli et al. (2016). Further research is needed to understand the underlying mechanisms of relying on visual strategies, regardless of the hand’s laterality, in older adults.

It’s worth noting that these results May have been influenced by the decline in mental imagery often associated with aging. Specific aspects of imagery, such as image regeneration and manipulation, tend to deteriorate in mental-visual images as individuals age, which can lead to increased errors when implicitly rotating hands (Dror and Kosslyn, 1994). Furthermore, it’s important to acknowledge that age can influence various processes, including but not limited to a slowdown in cognitive functioning (Salthouse, 1996), deficits in spatial working memory (Salthouse, 1988), neural changes (Zapparoli et al., 2016), and impairments in motor performance (Buckles, 1993).

### Mannequin hands vs. real hands

Despite an overall improvement in both response time and accuracy when compared to Experiment 1 for palm-view stimuli, an ineffective approach was noted for the back 180° orientation, leading to error rates approaching 40%. This suggests that as the task became more complex (i.e., with an increased angle of rotation), participants experienced greater difficulty (Saimpont et al., 2009). When participants simulated mannequin hands, the lack of visual cues such as hand creases likely presented a greater challenge in processing palm-view stimuli. However, the inclusion of additional visual cues seemed to enhance participants capacity to discern between back and palm views. While real hands may have facilitated easier view distinction, the argument that participants perform worse for complex hand orientations is still supported. While the present experiment cannot directly compare mannequin and realistic hand stimuli used, a similar pattern in participant performance is still evident. Overall, the findings of this study are in line with the *Motor Simulation Theory*, suggesting that individuals follow the same motor principles and biomechanical constraints of the represented movement (Decety et al., 1989; Gentili et al., 2004; Jeannerod and Decety, 1995).

### Additional considerations

Our everyday lives are continuously enriched by action simulations that allow us to recall past events, anticipate potential actions, and seek additional information about the feasibility of those actions (Annett, 1995). The present study offers new insights into how action simulation strategies differ and how these action change over time. We acknowledge that the altered changes observed in the strategies employed during the HLJT can also be attributed to other age-related cognitive declines (for review see Seidler et al., 2010). The next logical step would be to do more cognitive screening in older adults to further confirm the results of this study.

In Experiment 1, the presentation of gray-colored depictions of mannequin hands from both back and palm views may have compromised the participants’ ability to detect differences between the two views, especially in difficult orientations. However, in Experiment 2, we employed real hands to facilitate the determination of both the back and palm viewpoints. At present, there has been no study examining how the choice of hand-related stimuli in the HLJT may affect response patterns and accuracy. Our lab plans to conduct a comparative analysis of various hand stimuli to determine if one specific stimulus outperforms the others. Furthermore, several studies have demonstrated that the back of the hand employs visual strategies (i.e., a third-person perspective), while the palm of the hand employs kinesthetic motor strategies (i.e., a first-person perspective; Bläsing et al., 2013; Gentilucci et al., 1998; Nagashima et al., 2021). However, there is still a need to explore why older adults transition between an adaptive and non-adaptive strategy for specific viewpoints and orientations. Our lab plans to explore other objective measures such as eye-gaze dynamics and pupillometry during motor imagery that may provide novel insights into human internal action simulations. In particular, the role of vision may reveal several aspects of behavior patterns that occur during simulation that have not been examined.

The results of this study suggest that proprioceptive input (i.e., the on-line position of the participant’s hand) is critical to action simulation, especially when viewing hands from the palm. MOLA effects observed for palm-view stimuli may reflect participants using the configuration of their hands placed on the keyboard to align their hands with visually presented stimuli. Furthermore, it is possible that the results reported here are influenced by age-related declines in proprioception (Herter et al., 2014; Skinner et al., 1984) as these abilities are essential to correctly identifying the laterality of body parts. The effects of peripheral factors, such as body position, on motor imagery performance should be explored in aging populations. Future research is required to assess whether the adopted hand posture influences response times and accuracy when implicitly rotating hands in varying orientations and views.

## Conclusion

In conclusion, this study provides evidence for a decline in implicit motor imagery among older adults when solving the laterality judgment task. In particular, our study suggests that when simulating typical or challenging upper limb movements from the back or palm of the hands, different strategies (e.g., motoric or visual strategies) May be used depending on one’s age. However, as one ages, a greater decline in kinesthetic motor imagery over visual motor imagery is seen. To confirm the results obtained in this study, further cognitive screening in older adults will be required.

Based on the similarities between implicit motor imagery and actual movements, this study will offer insights into its application as a tool for enhancing motor performance in aging populations. Motor imagery has been shown to be an effective tool for a wide range of clinical populations and may be a useful addition to physiotherapy and occupational therapy as it allows older adults to safely simulate movements while reducing the physical demands of actual movement. Considering that cognitive mechanisms underlying motor imagery vary by age, implicit motor imagery should be used on an individual treatment basis rather than as a one-size-fits-all approach.

## Data Availability

The datasets presented in this study can be found in online repositories. The names of the repository/repositories and accession number(s) can be found at: Marotta, Jonathan, 2022, “The Effects of Implicit Motor Imagery in Aging using the Hand Laterality Judgment Task,” https://doi.org/10.34990/FK2/GWWSIM, Borealis, V1.
