# Supplementary material for: Implicit motor imagery: examining motor vs. visual strategies in laterality judgments among older adults
Source: Front Psychol. 2024 Oct 2;15:1445152. doi: 10.3389/fpsyg.2024.1445152 (PMC11481337; doi:10.3389/fpsyg.2024.1445152)
Supplement: Supplementary file 1 [file Data_Sheet_1.PDF]

Experiment 1: Mean Response Time and Standard Errors (ms)

Variables	Mean Response Time (ms)	Standard error (ms)
**View**	Back Mean= 2555 vs. Palm Mean= 2728	Back SE= 128 vs. Palm SD= 146
**Laterality**	Right Mean= 2680 vs. Left Mean= 2603	Right SE= 140 vs. Left SE= 130
**Orientation**	1. 0° Mean= 2287 vs. 90L° Mean= 2925<br>2. 0° Mean= 2287 vs. 180° Mean= 3158<br>3. 90M° Mean= 2195 vs. 90L° Mean= 2925<br>4. 90M° Mean= 2195 vs. 180° Mean= 3158<br>5. 90L° Mean= 2925 vs. 180° Mean= 3158	1. 0° SE =119 vs. 90L° SE= 175<br>2. 0° SE =119 vs. 180° SE= 162<br>3. 90M° SE=111 vs. 90L° SE= 175<br>4. 90M° SE=111 vs. 180° SE= 162<br>5. 90L° SE= 175 vs. 180° SE= 162
**Age**	Young Mean= 1879 vs. Old Mean= 2891	Young SE= 68.7 vs. Old SE= 81.4
**Laterality x Sex**	Right Male Mean= 2261 vs. Left Male Mean =2422	Right Male SE= 85.8 vs. Left Male SE= 81.4
**Laterality x Age**	1. Right Young Mean= 1867 vs. Right Old Mean= 2815<br>2. Right Young Mean= 1867 vs. Left Old Mean= 2967<br>3. Right Old Mean= 2815 vs. Left Young Mean= 1891<br>4. Left Young Mean= 1891vs. Left Old Mean= 2967	1. Right Young SE= 73 vs. Right Old SE= 86.5<br>2. Right Young SE= 73 vs. Left Old SE= 82.1<br>3. Right Old SE= 86.5 vs. Left Young SE= 69.2<br>4. Left Young SE= 69.2 vs. Left Old SE= 82.1
**View x Orientation**	1. Back 0° Mean= 1654 vs. Back 90M° Mean= 1977<br>2. Back 0° Mean= 1654 vs. Back 90L° Mean=2261<br>3. Back 0° Mean= 1654 vs. Back 180° Mean=3104<br>4. Back 0° Mean= 1654 vs. Palm 0° Mean=2462<br>5. Back 0° Mean= 1654 vs. Palm 90M° Mean=2032<br>6. Back 0° Mean= 1654 vs. Palm 90L° Mean=2977<br>7. Back 0° Mean= 1654 vs. Palm 180° Mean=2614<br>8. Back 90M° Mean= 1977 vs. Back 90L° Mean=2261	1. Back 0° SE= 34.3 vs. Back 90M° SE = 55.5<br>2. Back 0° SE = 34.3 vs. Back 90L° SE =68.3<br>3. Back 0° SE = 34.3 vs. Back 180° SE =94.5<br>4. Back 0° SE= 34.3 vs. Palm 0° SE= 71.6<br>5. Back 0° SE = 34.3 vs. Palm 90M° SE= 61.8<br>6. Back 0° SE =34.3 vs. Palm 90L° SE= 98.6<br>7. Back 0° SE = 34.3 vs. Palm 180° SE= 73.3<br>8. Back 90M° SE = 55.5 vs. Back 90L° SE =68.3

	9. Back 90M° Mean= 1977 vs. Back 180° Mean=3104<br>10. Back 90M° Mean= 1977 vs. Palm 0° Mean=2462<br>11. Back 90M° Mean= 1977 vs. Palm 90L° Mean=2977<br>12. Back 90M° Mean= 1977 vs. Palm 180° Mean=2614<br>13. Back 90L° Mean=2261 vs. Back 180° Mean=3104<br>14. Back 90L° Mean=2261 vs. Palm 90M° Mean=2032<br>15. Back 90L° Mean=2261 vs. Palm 90L° Mean=2977<br>16. Back 90L° Mean=2261 vs. Palm 180° Mean=2614<br>17. Back 180° Mean=3104 vs. Palm 0° Mean=2462<br>18. Back 180° Mean=3104 vs. Palm 90M° Mean=2032<br>19. Back 180° Mean=3104 vs. Palm 180° Mean=2614<br>20. Palm 0° Mean=2462 vs. Palm 90M° Mean= 2032<br>21. Palm 0° Mean=2462 vs. Palm 90L° Mean=2977<br>22. Palm 90M° Mean= 2032 vs. Palm 90L° Mean=2977<br>23. Palm 90M° Mean= 2032 vs. Palm 180° Mean=2614<br>24. Palm 90L° Mean=2977 vs. Palm 180° Mean=2614	9. Back 90M° SE = 55.5 vs. Back 180° SE =94.5<br>10. Back 90M° SE = 55.5 vs. Palm 0° SE= 71.6<br>11. Back 90M° SE = 55.5 vs. Palm 90M° SE= 61.8<br>12. Back 90M° SE = 55.5 vs. Palm 180° SE= 73.3<br>13. Back 90L° SE =68.3 vs. Back 180° SE =94.5<br>14. Back 90L° SE =68.3 vs. Palm 90M° SE= 61.8<br>15. Back 90L° SE =68.3 vs Palm 90L° SE= 98.6<br>16. Back 90L° SE =68.3 vs Palm 180° SE= 73.3<br>17. Back 180° SE =94.5 vs. Palm 0° SE= 71.6<br>18. Back 180° SE =94.5 vs. Palm 90M° SE= 61.8<br>19. Back 180° SE =94.5 vs. Palm 180° SE= 73.3<br>20. Palm 0° SE= 71.6 vs. Palm 90M° SE= 61.8<br>21. Palm 0° SE= 71.6 vs. Palm 90L° SE= 98.6<br>22. Palm 90M° SE= 61.8 vs. Palm 90L° SE= 98.6<br>23. Palm 90M° SE= 61.8 vs. Palm 180° SE= 73.3<br>24. Palm 90L° SE= 98.6 vs. Palm 180° SE= 73.3

Experiment 2: Mean Response Time and Standard Errors (ms)

Variables	Mean Response Time (ms)	Standard error (ms)
**Orientation**	1. 0° Mean= 2287 vs. 90L° Mean=2925<br>2. 0° Mean= 2287 vs. 180° Mean = 3158°<br>3. 90M° Mean= 2195 vs. 90L° Mean=2925<br>4. 90M° Mean= 2195 vs. 180° Mean = 3158°	1. 0° SE= 119 vs. 90L° SE=175<br>2. 0° SE= 119 vs. 180° SE=162<br>3. 90M° SE= 111 vs. 90L° SE=175<br>4. 90M° SE= 111 vs. 180° SE=162
**View x Orientation**	1. Back 0° Mean= 1915 vs. Back 90M° Mean= 2245<br>2. Back 0° Mean= 1915 vs. Back 90L° Mean= 2629<br>3. Back 0° Mean= 1915 vs. Back 180° Mean= 3431<br>4. Back 0° Mean= 1915 vs. Palm 0° Mean= 2659<br>5. Back 0° Mean= 1915 vs. Palm 90L° Mean= 3221<br>6. Back 0° Mean= 1915 vs. Palm 180° Mean= 2884<br>7. Back 90M° Mean= 2245 vs. Back 90L° Mean= 2629<br>8. Back 90M° Mean= 2245 vs. Back 180° Mean= 3431<br>9. Back 90M° Mean= 2245 vs. Palm 90L° Mean= 3221<br>10. Back 90M° Mean= 2245 vs. Palm 180° Mean= 2884<br>11. Back 90L° Mean= 2629 vs. Back 180° Mean= 3431<br>12. Back 90L° Mean= 2629 vs. Palm 90M° Mean= 2145<br>13. Back 180° Mean= 3431 vs. Palm 0° Mean= 2659<br>14. Back 180° Mean= 3431 vs. Palm 90M° Mean= 2145<br>15. Back 180° Mean= 3431 vs. Palm 180° Mean= 2884<br>16. Palm 0° Mean= 2659 vs. Palm 90M° Mean= 2145<br>17. Palm 0° Mean= 2659 vs. Palm 90L° Mean= 3221	1. Back 0° SE= 105 vs. Back 90M° SE = 117<br>2. Back 0° SE= 105 vs. Back 90L° SE = 161<br>3. Back 0° SE= 105 vs. Back 180° SE = 181<br>4. Back 0° SE= 105 vs. Palm 0° SE= 169<br>5. Back 0° SE= 105 vs. Palm 90L° SE= 232<br>6. Back 0° SE= 105 vs. Palm 180° SE= 160<br>7. Back 90M° SE =117 vs. Back 90L° SE = 161<br>8. Back 90M° SE =117 vs. 180° SE = 181<br>9. Back 90M° SE =117 vs. Palm 90L° SE= 232<br>10. Back 90M° SE =117 vs. Palm 180° SE= 160<br>11. Back 90L° SE = 161 vs. Back 180° SE = 181<br>12. Back 90L° SE = 161 vs. Palm 90M° SE= 113<br>13. Back 180° SE = 181 vs. Palm 0° SE= 169<br>14. Back 180° SE = 181 vs. Palm 90M° SE= 113<br>15. Back 180° SE = 181 vs. Palm 180° SE= 160<br>16. Palm 0° SE= 169 vs. Palm 90M° SE= 113<br>17. Palm 0° SE= 169 vs. Palm 90L° SE= 232

	18. Palm 90M° Mean= 2145 vs. Palm 90L° Mean= 3221<br>19. Palm 90M° Mean= 2145 vs. Palm 180° Mean= 2884	18. Palm 90M° SE= 113 vs. Palm 90L° SE= 232<br>19. Palm 90M° SE= 113 vs. Palm 180° SE= 160
**Laterality x View x Age**	1. Right Palm Young Mean = 2692 vs. Left Back Young Mean= 2463<br>2. Right Palm Old Mean = 2844 vs. Left Back Old Mean= 2573<br>3. Left Back Old Mean= 2573 vs. Left Palm Old Mean= 3000	1. Right Palm Young SE= 217 vs. Left Back Young Mean= 178<br>2. Right Palm Old SE= 223 vs. Left Back Old SE= 183<br>3. Left Back Old SE= 183 vs. Left Palm Old Mean SE= 245
